# Transcriptomic characterization of short duration endoplasmic reticulum stress on cultured human proximal tubule cells

**DOI:** 10.1186/1471-2105-15-S10-P5

**Published:** 2014-09-29

**Authors:** Yan Zhang, Michelle Barati, Ignacio Munoz, Ming Li, Danny Wilkey, Eric Rouchka, Michael Merchant

**Affiliations:** 1 Department of Neuroscience Training, University of Louisville, KY 40202, USA; 2 Department of Medicine, University of Louisville, Louisville, KY 40202, USA; 3 Department of Computer Engineering and Computer Science, University of Louisville, Louisville, KY 40292, USA

## Background

Stress granules (SG) are formed as collections of protein and RNA (ribonucleoprotein structures) and continuously assembled/disassembled in response to stresses such as heat, osmotic, or oxidant stress; representing an attempt to survive the stress through salvage of important proteins and RNA. Recent research suggests diabetic nephropathy (DN) may change or alter SG biology and in conditions that model DN may involve the receptor for activated C-kinases (RACK1). The incorporation of RACK1 into stress granules may down-regulate programmed cell death and further may impart the ability to scaffold to and sequester key signaling proteins to affect cell survival or death. We hypothesized that the inappropriate or dysregulated scaffolding of proteins or RNA into stress granules may be of importance in the development of diabetic nephropathy. The goal of this study is to qualitatively and semi-quantitatively characterize the effects of cell culture conditions modeling diabetes and ER stress in conjunction with over-expression studies of SG stabilizing proteins on RNA transcripts.

## Materials and methods

### Cell culture

Immortalized human proximal tubule cells (HK2) were grown to 70-80% confluence, transfected with GFP, GFP-RACK1, or GFP-dnRACK1 for 24hr and then treated with 0.5uM thapsigargin (Tg) or vehicle (DMSO) for 30min prior to cell lysis and sample collection.

### Density gradient ultracentrifugation (UC)

Protein lysate (500ug) was fractionated using Optiprep (Sigma Aldrich) density gradient medium to prepare 1+7 layered fractions, containing 2.5% Optiprep plus the 500ug lysate and 5, 10, 15, 20, 25, 50, and 54% Optiprep, into thick walled UC tubes, loaded into a SW 55 Ti rotor and spun at 40.6K rpm for 3hr at 4C (medium acceleration and no brake). Fractions were collected and used for immunoblot analysis and also transcriptomic analyses.

### Western blotting

Localization of marker proteins to specific UC fractions was achieved by immunoblot analysis for GFP and RACK1 (transfection markers), TIA-1 or G3BP (stress granule markers), and GADPH.

### Transcriptomics

Total RNA was isolated from UC fractions using TRIzol reagent (Life Technologies, Carlsbad, CA) and yields quantified using either 260/280 reading using a Nanodrop 1000 (Thermo Scientific, Wilmington, DE) or Qubit fluorometric quantification (Life Technologies, Carlsbad, CA). The quality of isolated RNA per UC fraction was assessed using an Agilent 2100 Bioanalyzer. For RNAseq analysis, the samples were sequenced with a 50 bp single-end run on an Illumina GAIIx system at Cofactor Genomics (St. Louis, MO). Bioinformatics analysis was conducted at the Kentucky Biomedical Research Infrastructure Network (KBRIN) Bioinformatics Core. Reads passed quality control were aligned to *Homo sapiens* reference genome (GenBank Assembly ID: GCA_000001405.1) using Tophat (version 2.0.4). Assembly was performed using Cufflinks (version 2.1.1) resulting in genes with significantly differential expression. Functional analysis was then conducted by MetaCore (Thomson Reuters Inc., Carlsbad, CA).

## Results

Analysis of HK2 cell lysates fractionated using Optiprep density gradient UC demonstrated by immunoblot that cytoplasmic proteins were found in the 2.5% and 5% Optiprep fractions while SG markers (TIA1 or G3BP) and RACK1/dnRACK1 constructs were contained in the 5-15% Optiprep fractions. Total RNA yields ranges from the 10-400ng/uL range with the highest yields found in the 50-54% Optiprep fractions. Qualitative analysis of RNA by the Agilent 2100 Bioanalyzer suggests that the 2.5-15% Optiprep fractions selectively contain non-ribosomal RNA while the 20-54% Optiprep fractions contain largely ribosome RNA. Qualitative comparison of RNAseq data suggests the RNA isolated from the 2.5% Optiprep fraction has more degradation than RNA isolated from 5% or 10% Optiprep fractions. As compared to DMSO (vehicle) treatment, Tg treatment induces greater that 2-fold changes (p-value <0.05) in the abundances of 1,300-1,500 genes in each transfection condition. These differences include lincRNA, antisense RNA, miRNA, and coding RNA transcripts. The strongest difference for Tg+RACK1 transfections was for an 80+ fold increase in the abundance of the coding RNA for CERCAM (ER luminal protein). The strongest difference for Tg+dnRACK1 transfections was for a 300+ fold decrease in the abundance of the coding RNA for KRAP (actin interacting protein regulating IP3R-mediated Ca++ release). The strongest difference for Tg+GFP transfections was for a generalized increase in the abundance of the coding RNA for histone H2 and H3 cluster variants. These results suggest that RNA abundance both qualitatively and quantitatively is affected by ER stress and the abundances may be modifiable by the transfection of proteins that can stabilize SG.

